# Circulating Tfh1 (cTfh1) cell numbers and PD1 expression are elevated in low-grade B-cell non-Hodgkin’s lymphoma and cTfh gene expression is perturbed in marginal zone lymphoma

**DOI:** 10.1371/journal.pone.0190468

**Published:** 2018-01-02

**Authors:** Elliot T. Byford, Matthew Carr, Eleni Ladikou, Matthew J. Ahearne, Simon D. Wagner

**Affiliations:** Leicester Cancer Research Centre and Ernest and Helen Scott Haematology Research Institute, University of Leicester, Leicester, United Kingdom; Institut Cochin, FRANCE

## Abstract

CD4^+^ T-cell subsets are found in the tumour microenvironment (TME) of low-grade B-cell non-Hodgkin’s lymphomas such as marginal zone lymphoma (MZL) or follicular lymphoma (FL). Both numbers and architecture of activating follicular helper T-cells (Tfh) and suppressive Treg in the TME of FL are associated with clinical outcomes. There has been almost no previous work on CD4^+^ T-cells in MZL. It is now recognised that circulating CD4^+^CXCR5^+^ T-cells are the memory compartment of Tfh cells. We determined differences in number of circulating Tfh (cTfh) cells and cTfh subsets between normal subjects and patients with FL or MZL. Lymphoma patients showed increased numbers of cTfh1 and reduced cTfh17 cells due to decreased expression of the subset-defining marker CCR6 in patients. PD1, a surface marker associated with Tfh cells, showed increased expression on cTfh subsets in patients. Focusing on MZL we determined expression of 96 T-cell associated genes by microfluidic qRT-PCR. Analysis of differentially expressed genes showed significant differences between normal subjects and patients both for bulk cTfh (CCL4) and the cTfh1 subset (JAK3). While our findings require confirmation in larger studies we suggest that analysis of number and gene expression of circulating T-cells might be a source of clinically useful information as is the case for T-cells within lymphoma lymph nodes.

## Introduction

The tumour microenvironment (TME) in B-cell non-Hodgkin’s lymphomas (B-NHL) contains T-cells, stromal cells and humoral factors such as cytokines and chemokines. The TME is essential for supporting the proliferation and survival of lymphoma cells and in resisting the effects of chemotherapy. Interrupting the signalling pathways mediated by cells or humoral factors might enhance the effects of chemotherapy and suggests that the TME is a target for therapy[1,2].

Both numbers and architecture of CD4^+^ T-cells in the TME of low-grade B-NHL such as follicular lymphoma (FL) are associated with clinical outcome[3–6]. The follicular helper (Tfh) T-cell subset has been a focus of particular interest in both follicular lymphoma [7] and chronic lymphocytic leukaemia (CLL) [8–10] in part because cytokines produced by Tfh cells drive proliferation of malignant B-cells[6,8,9]. The pathogenesis of other low-grade B-NHLs, extranodal marginal zone lymphoma (MZL) of mucosa-associated lymphoid tissue (MALToma) are directly related to abnormal immune responses that can be driven by a variety of micro-organisms [11,12].

Tfh cells are located in germinal centres and are required for high affinity antibody responses in normal immunity [13]. However, germinal centre function is regulated not only by Tfh cells but also by suppressive follicular regulatory (Tfr) T-cells[14,15]. Tfh and Tfr cells are characterised by surface expression of CD4, CXCR5 and PD1 with nuclear expression of BCL6 but only Tfr cells express the transcription factor FOXP3.

Peripheral blood populations of CD4^+^CXCR5^+^ cells have been identified [16] and represent circulating memory compartments of Tfh cells [17,18] or Tfr cells [19]. Importantly circulating CD4^+^CXCR5^+^PD1^hi^CCR7^lo^ T-cells reflect active Tfh differentiation in lymphoid organs [18] and their numbers in peripheral blood correlate with clinical measures of disease activity in autoimmunity. Peripheral blood Tfh subsets have, therefore, been postulated to be biomarkers, which will be potentially useful in monitoring response to treatment in autoimmunity, but there is little descriptive data in low-grade B-NHL although, in this context, they may reflect Tfh in the TME.

Populations of circulating CD4^+^CXCR5^+^ cells have recently have been shown to be very heterogeneous[20]. One approach to understanding their heterogeneity has been to analyse the expression of the chemokine receptors, CXCR3 and CCR6 of CD4^+^CXCR5^+^ cells [21,22]. CXCR3^+^CCR6^-^ cells express the transcription factor TBX21 (also called T-bet) and produce interferon-γ, a Th1 cytokine, whereas CXCR3^-^CCR6^-^ cells express the transcription factor GATA3 and produce IL-4, IL-5, and IL-13, Th2 cytokines, while CXCR3^-^CCR6^+^ cells express the transcription factor RORγT and produce IL-17A and IL-22, Th17 cytokines. On this basis CD4^+^CXCR5^+^CXCR3^+^CCR6^-^ cells are called circulating Tfh1 (cTfh1), CD4^+^CXCR5^+^CXCR3^-^CCR6^-^ are cTfh2 and CD4^+^CXCR5^+^CXCR3^-^CCR6^+^ are cTfh17 [20]. There has been little work on the remaining population CD4^+^CXCR5^+^CXCR3^+^CCR6^+^ but conventional helper T-cells, CD4^+^CXCR5^-^, which are transitioning between Th17 and Th1 demonstrate CXCR3^+^CCR6^+^ and are described as Th1/17 [23,24]. By analogy, therefore, in this work we refer to CD4^+^CXCR5^+^CXCR3^+^CCR6^+^ cells as cTfh1/17.

There are functional differences between the various cTfh subsets; cTfh2 and cTfh17 provide efficient B-cell help, largely through secretion of IL21, and are increased in many autoimmune disorders [20] and also high-grade B-NHL [25] whereas cTfh1 are less efficient helpers and are associated with the generally poor antibody responses to influenza vaccine [26]. These functional differences are potentially relevant to their roles in lymphoma: cTfh2 and cTfh17 might drive proliferation of lymphoma cells whereas the less efficient cTfh1 might be a feature of disease in which proliferation of lymphoma is more cell autonomous.

In neither MZL nor FL have perturbations in circulating Tfh (cTfh) been previously investigated. In this report we have for the first time determined the numbers of cTfh in the low-grade B-NHL, FL and MZL and surprisingly found significant differences in cTfh1. We have further compared gene expression between cTfh from patients with MZL and those of normal subjects to establish that there are differences, which require confirmation in larger studies.

## Materials and methods

### Patients

Peripheral blood samples were obtained from 12 healthy volunteers, 7 MZL patients, and 6 FL patients, 2 lymphoplasmacytic lymphoma patients and 1 low-grade B-cell non-Hodgkin’s lymphoma not otherwise specified (Table 1). Patients were recruited from September 2016 to March 2017 after having given informed, written consent. Patients had not received chemotherapy for at least 3 months before sample collection. Healthy volunteers were excluded if they had experienced illness or had been vaccinated within 3 weeks or taken medication within 7 days of sample collection. The study was approved by the University Hospitals of Leicester NHS Trust Research Ethics Committee (06/Q2501/122) and was carried out in accord with the principles of the Declaration of Helsinki. Samples were obtained from patients and healthy subjects after written informed consent.

**Table 1:** Patient characteristics.

Characteristic		Normal Subjects (n = 12)	MZL (n = 7)	BNHL (n = 9)
Age (Years)	Mean (Range)	33.3 (25–65)	71.9 (54–84)	63.7 (43–81)
	Number <40 (%)	9 (75%)		
	Number >40 (%)	3 (25%)	7 (100%)	9 (100%)
				
Gender	Male	7 (58%)	4 (57%)	5 (55%)
	Female	5 (42%)	3 (43%)	4 (45%)
				
Diagnosis	MZL		7	
	FL			6
	B-NHL-NOS			1
	LL			2
Clinical Stage	I		1	1
	II		1	1
	III		1	2
	IV		4	5

Patients were divided into two categories: marginal zone lymphoma (MZL) and low-grade B-cell non-Hodgkin’s lymphoma (BNHL). BNHL, in turn, includes follicular lymphoma (FL), lymphoplasmacytoid lymphoma (LL) and B-cell non-Hodgkin’s lymphoma not otherwise specified (BNHL-NOS). MZL cases comprised nodal MZL (n = 3), extra-nodal MZL (n = 3) and splenic marginal zone lymphoma (n = 1). All MZL cases were CD5^-^ and CD10^-^.

### Flow cytometry

CD4^+^ cells were isolated from the peripheral blood isolated by density gradient centrifugation using Ficoll-Paque Plus (GE Healthcare Life Sciences, 17-1440-02) in combination with RosetteSep^™^ Human CD4^+^ T Cell Enrichment Cocktail (STEMCELL Technologies, #15062). The efficacy of CD4^+^ isolation was routinely ≥ 94%. CD4^+^ cell suspensions were cryopreserved. Enumeration of CD4^+^ cells was not affected by cryopreservation as demonstrated by comparing the CD4^+^ purity of freshly isolated cells with those from the same blood sample that had been cryopreserved for 3 days. All flow cytometry data acquisition and cell sorting was performed on a BD FACSAria II (BD Biosciences, San Jose, USA) flow cytometer using BD FACSDiva 6.3.1 software. Two antibody panels were employed: Panel 1 was designed to include all markers necessary for the identification of cTfh cells and their subsets and Panel 2, which included FoxP3 in place of CXCR3 and CCR6, enabled the identification of Treg and cTfr (Table 2). A viability dye (LIVE/DEAD^®^ Fixable Near-IR Dead Cell Stain Kit, Thermofisher Scientific, #L10119) to exclude dead cells and the naive T-cell marker, CD45RA, were used in both panels.

**Table 2:** Antibodies employed for flow cytometry analysis and sorting.

Antibody Target	Conjugated Fluorophore	Host Species	Isotype	Clone	Manufacturer	Catalogue Number
**CD4**	BB515	Mouse	IgG1κ	RPA-T4	BD Biosciences	564419
**CD45RA**	APC-H7	Mouse	IgG2b κ	HI100	BD Biosciences	560674
**PD1**	PE	Mouse	IgG1 κ	MIH4	BD Biosciences	557946
**CXCR5**	BV421	Rat	IgG2b κ	RF8B2	BD Biosciences	562747
**CCR6**	BV711	Mouse	IgG1 κ	IIA9	BD Biosciences	563923
**CXCR3**	BUV395	Mouse	IgG1 κ	IC6	BD Biosciences	565223
**CXCR5**	BV711	Mouse	IgG1 κ	J252D4	Biolegend	356934
**FOXP3**	BV421	Mouse	IgG1 κ	206D	Biolegend	320124

Tonsil lymphocyte suspensions were employed to set gates for the identification of germinal centre Tfh cells, defined as CXCR5^hi^PD1^hi^. A threshold of 70–100% of the maximum fluorescence intensity for these markers was used. In peripheral blood, cTfh cells were identified as CD4^+^ CXCR5^+^ and the distribution of their subsets was elucidated[20]. The expression level for PD1^hi^ for detection of cTfh was the same as that used to identify tonsillar Tfh cells.

Treg in the blood were defined by CD4^+^CD45RA^-^FOXP3^hi^ as this is more specific than CD4^+^FOXP3^+^. To determine a robust definition for FOXP3 expression, we concatenated 11 healthy volunteer samples and set the threshold at 95% of the maximum fluorescence intensity of the FOXP3 marker: cTfr cells were identified as CD4^+^FOXP3^hi^CXCR5^+^.

### Visual stochastic neighbour embedding (ViSNE) analysis of flow cytometry data

Visual stochastic neighbour embedding (ViSNE) is an implementation of the Barnes Hunt t-Distributed stochastic neighbour embedding algorithm (t-SNE) in which an unsupervised, algorithmic approach is employed to automatically cluster phenotypically distinct populations of cells, by marker expression profiles, and display the results on a 2-dimensional plot [27]. Panel 1 data from healthy controls, MZL and FL patients were gated to live, CD45RA^-^CD4^+^CXCR5^+^ cells before being concatenated and uploaded to Cytobank Premium (https://www.cytobank.org/) as three separate fcs files. Fluorescence data channels were transformed to hyperbolic arcsine (arcsinh) scales with a scale argument of 50. 20000 events were down-sampled for each group, and the fluorescence parameters for CD4, CXCR5, PD1, CXCR3 and CCR6 were employed in the analysis, which was performed with 1000 iterations, a perplexity of 30, and a theta of 0.5.

### Microfluidic RT-qPCR

A custom panel of 96 genes (including 4 candidate reference genes) implicated in CD4^+^ T-cell biology (migration, differentiation, effector function and cell signalling pathways) was created, and primers designed using the D3 Assay Design service (Fluidigm, https://d3.fluidigm.com/) (S1 Table). Using the same staining protocol and gating strategy outlined above, 10 cells in triplicate were sorted from selected cTfh subsets of 5 normal subjects and 4 MZL patients. Cells were sorted directly into wells of a 96-well plate (Thermofisher Scientific #N8010560) containing reverse transcription reaction mix (5 μl) (0.25 μl 10% NP40 (ThermoFisher Scientific, #28324), 1.2 μl 5xVILO Reaction Mix (ThermoFisher Scientific, #1174050), 0.3 μl 20 U/μl SUPERase-In (ThermoFisher Scientific, #AM2694), 3.25 μl nuclease free water (Sigma-Aldrich, #W1754-1VL) and stored at -80°C. Samples were thawed, denatured at 65°C for 90 s and reverse transcriptase added (1 μl) (0.15 μl 10x SuperScript Enzyme Mix (ThermoFisher Scientific, #11754050), 0.12 μl T4 Gene 32 Protein (New England Biolabs Inc., #M0300S), 0.73 μl nuclease free water (Sigma-Aldrich, #W1754-1VL) followed by incubations in a thermal cycler with settings 25°C for 5 minutes, 50°C for 30 minutes, 55°C for 25 minutes, 60°C for 5 minutes and 70°C for 10 minutes.

cDNA (1.25 μl) was combined with a pre-amplification mix (3.75 μl) (0.5 μl "pooled" gene assay mix, 1 μl Pre-Amp Master Mix (Fluidigm, #100–5581) and 2.25 μl nuclease free water (Sigma-Aldrich, #W1754-1VL)) and samples were incubated in a thermal cycler at 95°C for 2 minutes followed by 20 cycles of 95°C for 15 s and 60°C for 4 minutes. The “pooled” gene assay mix was constructed by combining 1μl of all 96 primers (forward and reverse combined) with DNA suspension buffer (104 μl) (Teknova #T0223). Following pre-amplification unincorporated primers were removed by exonuclease I digestion (2 μl of 4 U/μl exonuclease I (New England Biolabs Inc., #M0293S) by incubation in a thermal cycler at 37°C for 30 minutes followed by 80°C for 15 minutes. Samples were then diluted 5-fold using 18 μl DNA suspension buffer (Sigma-Aldrich #W1754-1VL).

Sample mix (2.75 μl) (2X SSOFast EvaGreen Supermix with low ROX (2.5 μl) (BioRad #172–5211) and 20X DNA Binding Dye (0.25 μl) (Fluidigm #100–7609)) was added to the pre-amplified cDNA (2.25 μl). For each assay, primers (0.25μl) were combined with 2X Assay Loading Reagent (2.5 μl) (Fluidigm #100–7611) and DNA suspension Buffer (2.25 μl) (Sigma-Aldrich #W1754-1VL)) to produce an assay mix. Sample mix (5 μl) and assay mix (5 μl) were transferred to their respective inlets on the Integrated Fluidic Circuit of a Biomark HD system (Fluidigm, San Francisco, CA, USA). Data was acquired using the Biomark script “GE 96x96 PCR+Melt v2.pcl”. Ct values, amplification and melting curves for each reaction were visualised using Fluidigm Real-Time PCR Analysis software 4.1.3.

Using the software’s default settings, target amplification was detected for 5318 of 8064 reactions (65.95%). Ct values were exported into qBase+ 3.1 (Biogazelle) in which the GeNorm tool was used to identify ACTB, B2M and GAPDH as the most stably expressed reference genes of candidates across all samples. The expression fold change (EFC) for each reaction was calculated using the geometric mean of the Ct values of these three genes as a normalisation strategy to account for technical variation between reactions. EFC was scaled according to the combined average Ct values of the tonsil non-Tfh samples for analysing cTfh cells, or of the cTfh CXCR5^+^PD1^+^ samples for analysing tonsil samples.

Log transformed EFC values were exported into Multiple Experiment Viewer 4.9.0 and mean centered. Hierarchical clustering on genes and samples was carried out using the Pearson Correlation distance metric (average linkage clustering).

Several quality control measures were implemented at various steps of the analysis to exclude potentially unreliable Ct values. These were 1) Ct >25, 2) abnormal amplification curves, 3) abnormal melting curves, 4) variable expression of reference genes, 5) technical replicate variability. As a control experiment to ensure validity of our test results gene expression in tonsillar Tfh and non-Tfh cells was compared. Clusters of genes known to be important for Tfh cell function (CD40LG, CXCR5, IL-21, SH2DIA, CXCL13, CD84) or differentiation (IRF4, TIGIT, MTOR, VAV1) were detected.

### Statistics

Statistical analysis was performed using GraphPad Prism 7.00 (GraphPad Software, La Jolla California USA, http://www.graphpad.com) for flow cytometry data or Multiple Experiment Viewer 4.9.0 for gene expression data. Groups were compared using Mann- Whitney U tests. All statistical tests were two-tailed. An alpha level of 0.05 was considered significant.

## Results

### Total cTfh and cTfh subsets

**Fig 1:**
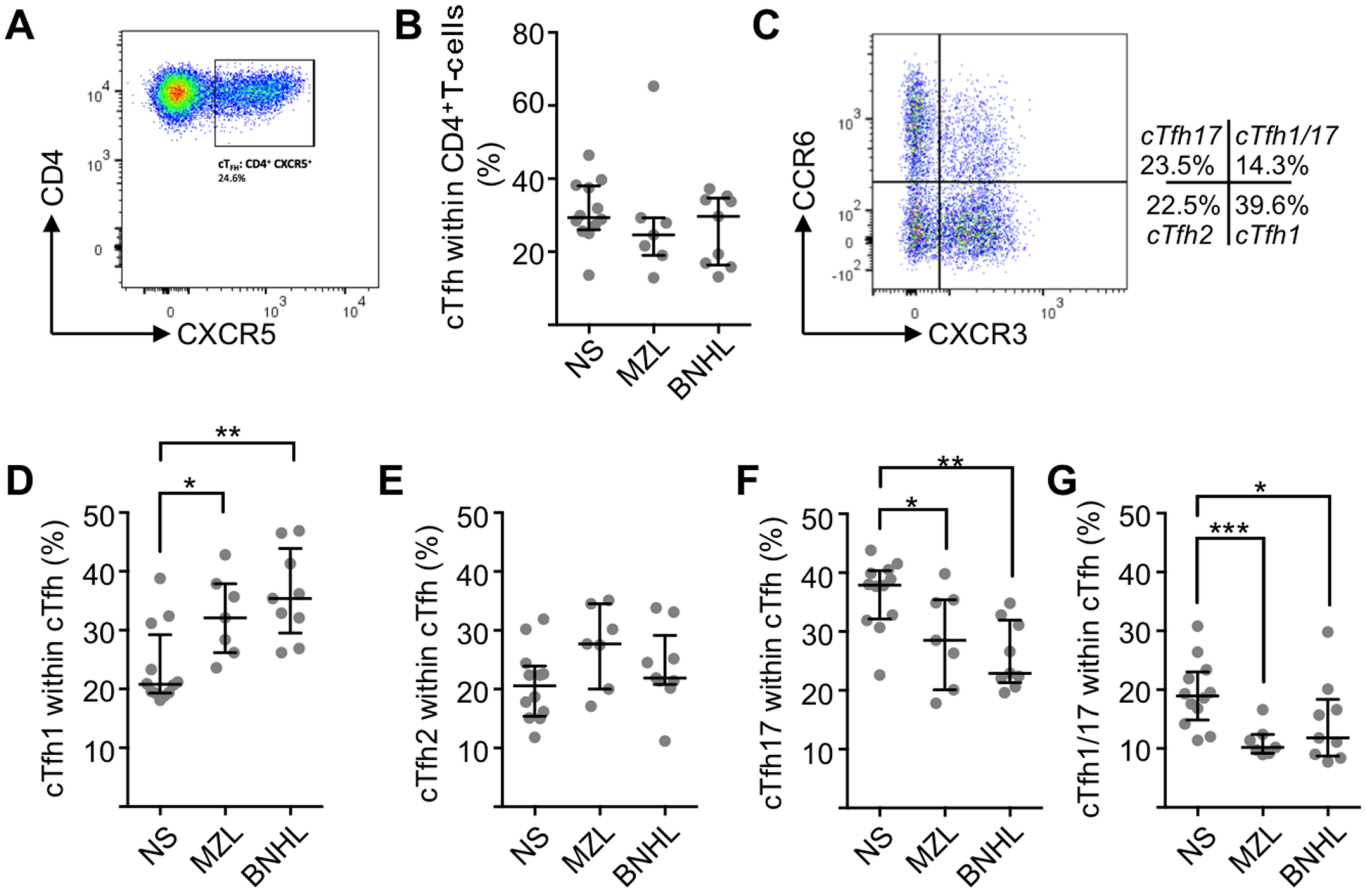
cTfh and cTfh subset proportions in normal subjects and low-grade B-NHL. **(A)** After lymphocyte gating and exclusion of doublets, live and CD45RA^-^ cells were identified for further analysis. The biaxial plot shows CXCR5 and CD4 expression from a representative MZL patient. The gate defines CD4^+^CXCR5^+^ (cTfh) cells as being 24.6% of total CD4^+^ T-cells. **(B)** cTfh cells as a percentage of CD4^+^ cells in normal subjects (median±SD, 18.9±5.7) and patients with FL (11.8±7.1) and MZL (10.2±2.7). Horizontal lines represent the median and bars represent inter-quartile range. **(C)** Biaxial flow cytometry plot showing CXCR3 and CCR6 expression on CD4^+^CD45RA^-^CXCR5^+^ cells. Four populations are identifiable: CXCR3^+^CCR6^-^ (cTfh1), CXCR3^-^CCR6^-^ (cTfh2), CXCR3^-^CCR6^+^ (cTfh17) and CXCR3^+^CCR6^+^ (cTfh1/17). **(D)** cTfh1 cells as a percentage of total cTfh cells. Medians are significantly (Mann-Whitney U-test) different between normal subjects (n = 12, 20.8±6.7%) and lymphoma patients (MZL, n = 7, 32.1±6.8%, *P* = 0.013 and FL, n = 9, 35.4±7.6%, *P* = 0.0056). **(E)** cTfh2 cells as a percentage of total cTfh cells. There are no significant differences between groups. **(F)** cTfh17 cells as a percentage of total cTfh cells. Medians are significantly different between normal subjects (37.9±5.9%) and lymphoma patients (MZL 28.5±8.2%, *P* = 0.045 and FL 22.9±5.7%, *P* = 0.0006). **(G)** cTfh1/17 cells as a proportion of total cTfh cells. Medians are significantly different between normal subjects (18.9±5.7%) and lymphoma patients (MZL 10.2±2.7%, *P* = 0.0008 and FL 11.8±7.1%, *P* = 0.016).

We defined cTfh as CD4^+^CD45RA^-^CXCR5^+^ and identified cells with this phenotype in the peripheral blood of normal subjects (n = 12) (mean±SEM; 31.0±2.5%) (Fig 1A). There was no overall difference in cTfh, as a proportion of total CD4^+^ T-cells, between normal subjects and those with MZL (n = 7) or B-NHL (n = 9) (Fig 1B).

Next we determined changes to cTfh subsets defined by CXCR3 and CCR6 (Fig 1C). In normal subjects cTfh1 were 23.6±1.9% (mean±SEM;) as a proportion of total cTfh, cTfh2 were 20.7±1.8 and cTfh17 were 36.3±1.7% while the poorly characterised CXCR3^+^CCR6^+^ population, which we designate cTfh1/17, were 19.3±1.6% (Fig 1D to 1G). Patients with lymphoma showed statistically significant increases in cTfh1 (Mann-Whitney U-test; MZL *P* = 0.01 and BNHL *P* = 0.005) and corresponding reduction in cTfh17 (MZL *P* = 0.04 and BNHL *P* = 0.0006) and cTfh1/17 (MZL *P* = 0.0008 and FL *P* = 0.02). Patients with lymphoma showed some increase in mean cTfh2 but this was not significant. Overall there were increased proportion of cTfh1 with reduced cTfh17 and cTfh1/17 in patients with MZL or BNHL.

### PD1 expression

**Fig 2:**
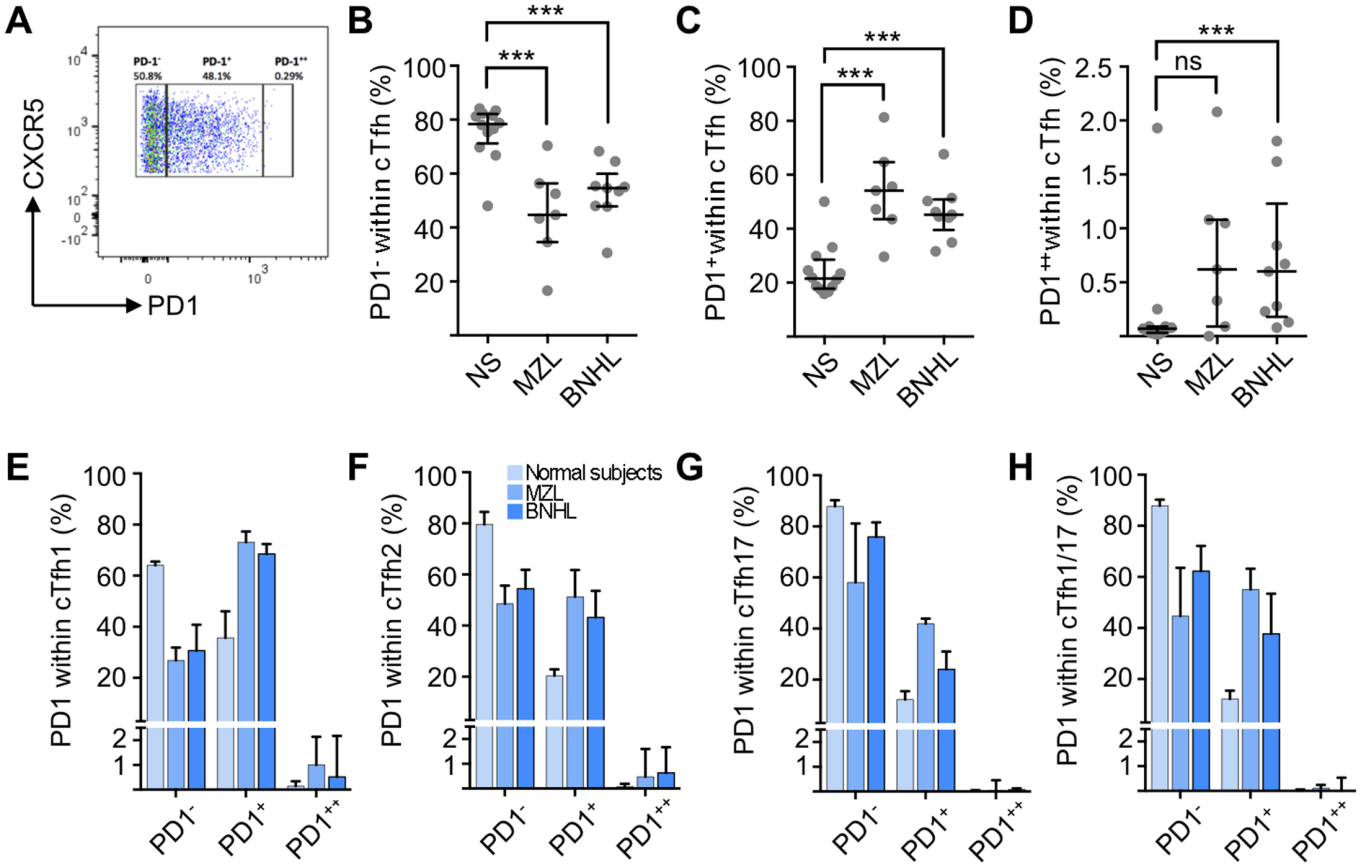
PD1 expression on cTfh subsets. **(A)** Biaxial flow cytometry plot showing expression of PD1 and CXCR5 on cells gated for CD4^+^CD45RA^-^CXCR5^+^. Gates defining PD1^++^, PD1^+^ and PD1^-^ cells were set by employing human tonsillar T-cells as controls. This representative example from a patient with MZL shows PD1^-^ 50.8%, PD1^+^ 48.1% and PD1^++^ 0.29%. **(B)** PD1^-^ cells as a proportion of total cTfh cells. Horizontal lines represent the median and bars represent inter-quartile range. Medians are significantly (Mann-Whitney U-test) different between normal subjects (n = 12, 78.4% (interquartile range 71.2 to 82.1%) and lymphoma patients (MZL n = 7, 44.7% (34.6 to 56.4%), *P* = 0.0008 and FL n = 9, 54.6% (47.9 to 60%), *P* = 0.0016). **(C)** PD1^+^ cells as a proportion of total cTfh cells. Medians are significantly different between normal subjects (21.5%, interquartile range 17.8 to 28.6%) and lymphoma patients (MZL 54.1% (43.5 to 64.7%), *P* = 0.0008 and FL 45.2% (39.5 to 50.9%), *P* = 0.0003). **(D)** PD1^++^ cells as a proportion of total cTfh cells. There is no significant difference between normal subjects (median 0.07%, interquartile range 0.03 to 0.09%) and MZL patients (median 0.62%, interquartile range 0.09 to 1.08%, *P* = 0.05) but there is a significant difference for BNHL (median 0.6%, interquartile range 0.18 to 1.23%, *P* = 0.003). Distribution of PD1 expression (PD1^++^, PD1^+^ or PD1^-^) within **(E)** cTfh1 cells, **(F)** cTfh2 cells, **(G)** cTfh17 cells and **(H)** cTfh1/17 cells.

cTfh helper capacity increases in line with PD1 expression [20] and, therefore, we determined the fraction of cTfh cells (CD4^+^CD45RA^-^CXCR5^+^) that were PD1^-^, PD1^+^ or PD1^++^. This is important because the level of PD1 expression correlates with Tfh activity and, therefore, is a surrogate measure of function[18,20,28]. The expression level for PD1^++^ was set with the same gating strategy as employed to identify germinal centre Tfh in tonsil (Fig 2A). As compared to normal subjects PD1^-^ cells were a reduced proportion of total cTfh cells in MZL (Mann-Whitney U-test; *P* = 0.0008) and FL (*P* = 0.0016) patients while PD1^+^ (MZL *P* = 0.0008 and FL *P* = 0.0013) and PD1^++^ (MZL not significant FL *P* = 0.013) were increased (Fig 2B to 2D). When analysed by cTfh subset (Fig 2E to 2H) the proportion of PD1^-^ cells was reduced in cTfh1, cTfh2, cTfh17 and cTfh1/17 while PD1^+^ cells were increased in all these subsets in lymphoma patients. Increased proportion of PD1^++^ appeared to be confined to cTfh1 and cTfh2 cells (Fig 2E and 2F). Therefore, patient samples demonstrate, for all CD4^+^CXCR5^+^ cells and for each cTfh subset, increased numbers of PD1^+^ cells and reduced numbers of PD1^-^ cells suggestive of increased helper T-cell function.

### Unbiased approach to analysis of cTfh subsets

**Fig 3:**
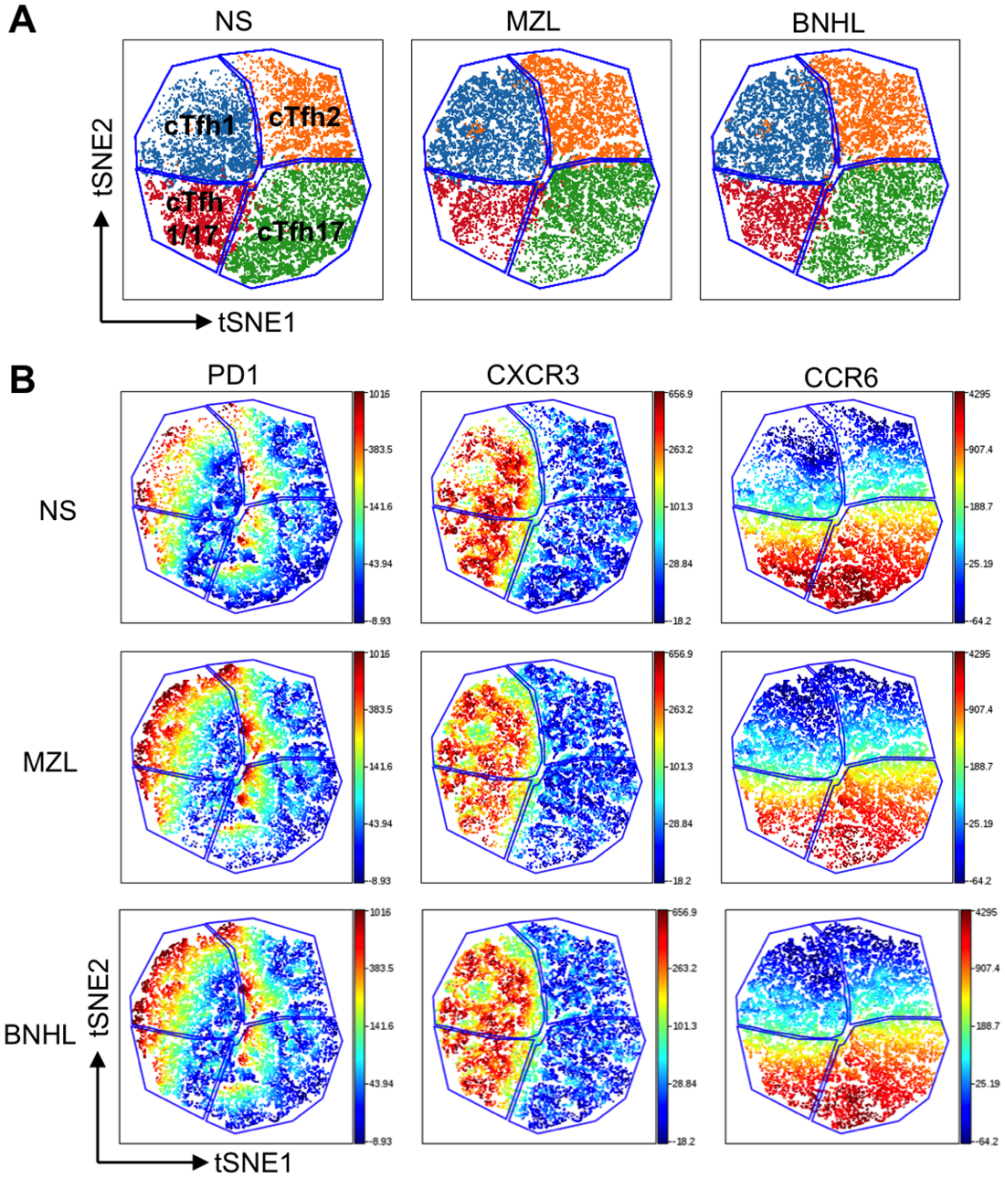
Clustering by ViSNE reveals surface marker expression changes in cTfh subsets. **(A)** Comparison of VisNE maps and gating from biaxial flow cytometry plots. Areas within blue lines are derived from the ViSNE maps. Cells coloured according to biaxial plots fro CXCR3 and CCR6 are coloured as shown: cTfh1 (blue), cTfh2 (orange), cTfh17 (green), cTfh1/17 (red). **(B)** The overall ViSNE map generated for each group is coloured according to the fluorescence intensity of the PD1, CXCR3 and CCR6 channels. Cells were pre-gated to cTfh. Level of expression is according to the bar to the right of the figure with red being highest expression.

In order to integrate all of the flow cytometry data and to increase confidence in the gating strategy, we employed ViSNE. t-SNE was applied to the CD4^+^CD45RA^-^CXCR5^+^ dataset and a very good correspondence between the ViSNE map gated on CXCR3 and CCR6 expression (blue lines in Fig 3A) and the individual cells coloured according to the gating of biaxial flow cytometry plots was obtained.

**Fig 4:**
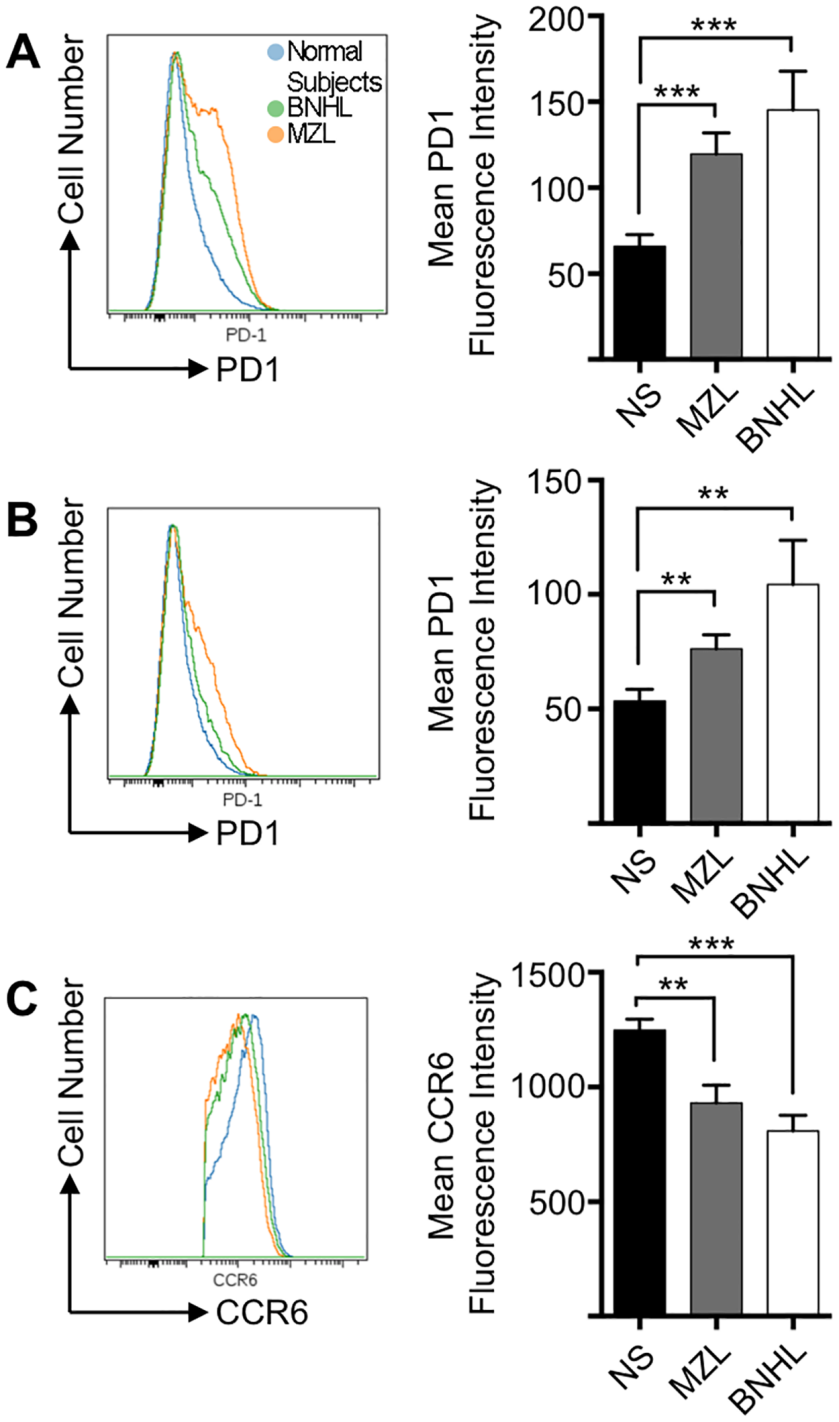
CCR6 and PD1 expression in cTfh subsets. **(A)** Concatenated FACS histogram showing superimposed PD1 expression in CD4^+^CXCR5^+^ cells from normal subjects (n = 12) and patients with MZL (n = 7) and BNHL (n = 9). The right hand column chart demonstrates PD1 expression (geometric mean fluorescence intensity) for each group (mean±SEM). There are significant increases in PD1 expression in MZL (Mann-Whitney U-test; *P* = 0.0008) and BNHL (*P* = 0.0005). **(B)** FACS histograms showing PD1 expression in CD4^+^CXCR5^+^CCR6^+^ cells from normal subjects and patients with MZL and BNHL. The right hand column chart demonstrates PD1 expression (geometric mean fluorescence intensity) for each group (mean±SEM). There are significant increases in PD1 expression in MZL (Mann-Whitney U-test; *P* = 0.0052) and BNHL (*P* = 0.0033). **(C)** FACS histograms showing CCR6 expression in CD4^+^CXCR5^+^CCR6^+^ cells from normal subjects and patients with MZL and BNHL. The right hand column chart demonstrates PD1 expression (geometric mean fluorescence intensity) for each group (mean±SEM). There are significant decreases in CCR6 expression in MZL (Mann-Whitney U-test; *P* = 0.0005) and BNHL (*P* = 0.0035).

Next we compared global changes in CXCR3, CCR6 and PD1 expression (Fig 3B) between normal subjects and patients with lymphoma. The heat maps show that sub-populations within cTfh1, cTfh2 and cTfh17 populations express high level PD1 in normal subjects and numbers in these sub-populations increase in lymphoma patients (Fig 3B). The most prominent changes are in the cTfh1 and cTfh17 subsets with only a minor fraction of cTfh2 expressing PD1. FACS histograms confirm significant increases in PD1 expression in the CD4^+^CXCR5^+^ (cTfh) cells (Fig 4A). CXCR3 and CCR6 are employed to define cTfh subsets. A similar analysis on CD4^+^CXCR5^+^CCR6^+^ (cTfh1/17 and cTfh17) populations also confirms increased PD1 expression in lymphoma patients (Fig 4B). Unexpectedly within the CD4^+^CXCR5^+^CCR6^+^ population there were significant reductions in CCR6 expression in lymphoma patients as compared to normal subjects (Fig 4C). Therefore, there are co-ordinated alterations to two surface markers (PD1 and CCR6) that appear to be specific to the circulating CD4^+^CD45RA^-^CXCR5^+^ T-cells of MZL and BNHL patients.

### Suppressive Treg and Tfr

**Fig 5:**
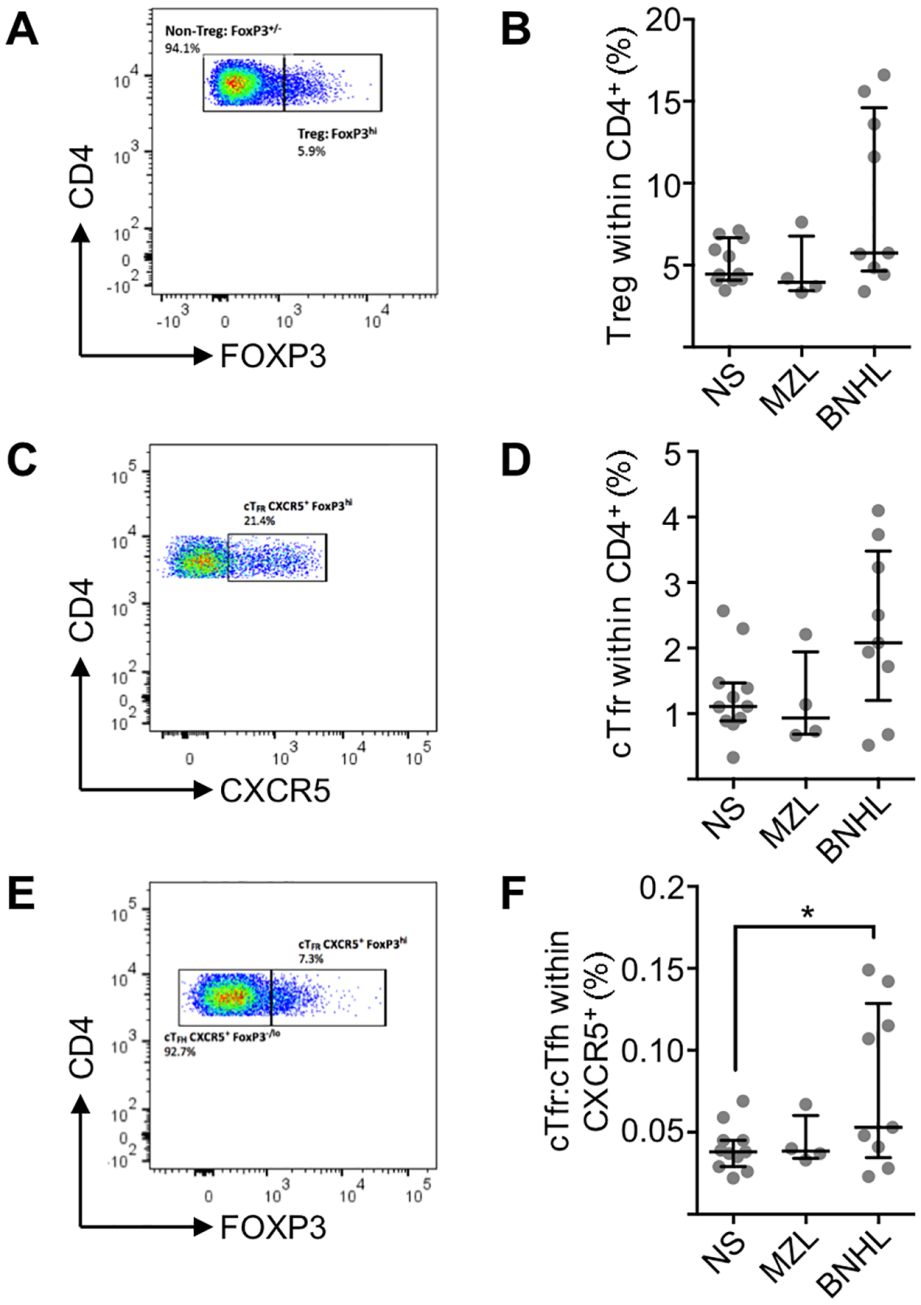
Suppressive Tregs and Tfr in normal subjects and low-grade lymphoma. **(A)** Biaxial flow cytometry plot showing expression of CD4 and FOXP3 on cells gated for CD4^+^CD45RA^-^. The highest 5% of cells from 11 concatenated NS were employed for the FOXP3^hi^ gate. This representative example from a patient with lymphoma shows Treg 5.9% of CD4^+^CD45RA^-^ cells. **(B)** Percentage of Treg within total CD4^+^ T-cells in normal subjects (n = 11), MZL (N = 4) and BNHL (n = 9). Horizontal lines represent the median and bars represent inter-quartile range. No significant differences were found between groups (Mann-Whitney U test). **(C)** Biaxial flow cytometry plot showing expression of CD4 and CXCR5 on cells gated for CD4^+^CD45RA^-^FOXP3^hi^. This representative example from a patient with lymphoma shows cTfr 21.4% of CD4^+^CD45RA^-^FOXP3^hi^ cells. **(D)** cTfr as a proportion of total CD4^+^ T-cells. Horizontal lines represent the median and bars represent inter-quartile range. There are no significant differences between normal subjects (n = 11) and patient with MZL (n = 4) or BNHL (n = 9). **(E)** Biaxial flow cytometry plot showing expression of CD4 and FOXP3 on cells gated for CD4^+^CD45RA^-^CXCR5^+^. Gates were set by employing human tonsillar T-cells as controls. This representative example from a patient with lymphoma shows cTfr (FOXP3^hi^) 7.3% and cTfh (FOXP3^-/lo^) 92.7% of CD4^+^CD45RA^-^CXCR5^+^ cells. **(F)** cTfr:cTfh ratio. Horizontal lines represent the median and bars represent inter-quartile range. BNHL patients (n = 9) showed a significant difference (Mann-Whitney U-test, *P* = 0.038) from normal subjects (n = 11) but MZL patients (n = 4) did not.

In order to complete the survey of peripheral blood CD4^+^ T-cell subsets we determined proportions of suppressive Treg and Tfr cells as proportions of total CD4^+^ T-cells (Fig 5A and 5C). Although there might be a group of BNHL patients with very high Treg numbers (Fig 5B) there was overall no significant change from normal subjects for either BNHL or MZL patients. A similar pattern is observed for cTfr cells (Fig 5C and 5D) and in line with this observation there was a significant correlation between Treg and cTfr numbers (R2 = 0.97, *P*<0.0001). cTfh reflect Tfh activity in lymph nodes [18] and while no such relationship has been determined for suppressive cTfr it has been demonstrated that changes in the ratio of Tfr:Tfh associate with alterations in immunity in mouse studies [29]. In normal subjects (n = 11) median cTfr:cTfh was 0.038 (interquartile range 0.029 to 0.045) and was not significantly different in patients with MZL (n = 4) (Fig 5E and 5F). However, largely due to a group of 4 FL cases with high cTfr:cTfh (range 0.1 to 0.15) compared to other FL cases (range 0.02 to 0.07), there is a significant difference (Mann-Whitney U-test; *P* = 0.038) from normal subjects. The data suggests that there can be perturbations of cTfr:cTfh in some FL patients.

### Gene expression differences in cTfh from patients and normal subjects

We have demonstrated that there are numerical differences in cTfh subsets between normal subjects and patients. In order to find out if there are accompanying differences in gene expression we carried out microfluidic qRT-PCR. This analysis was limited to 5 normal subjects and 4 MZL patients from the overall cTfh PD1^+^ population (CD4^+^CXCR5^+^PD1^+^) and the narrowed cTfh1 PD1^+^ (CD4^+^CXCR5^+^CXCR3^+^CCR6^-^PD1^+^) subset, because numbers of cTFh1 PD1^+^ cells were significantly increased in patients.

Firstly, sorted Tfh and non-Tfh cells from tonsil were obtained in order to compare gene expression and validate the procedures. Genes known to be important in Tfh function (CD40LG, CXCR5, IL21, SH2DIA, CXCL13 and CD84) or differentiation (BCL6, IRF4, TIGIT, MTOR and VAV1) showed increased expression in Tfh cells (S1 Fig).

**Fig 6:**
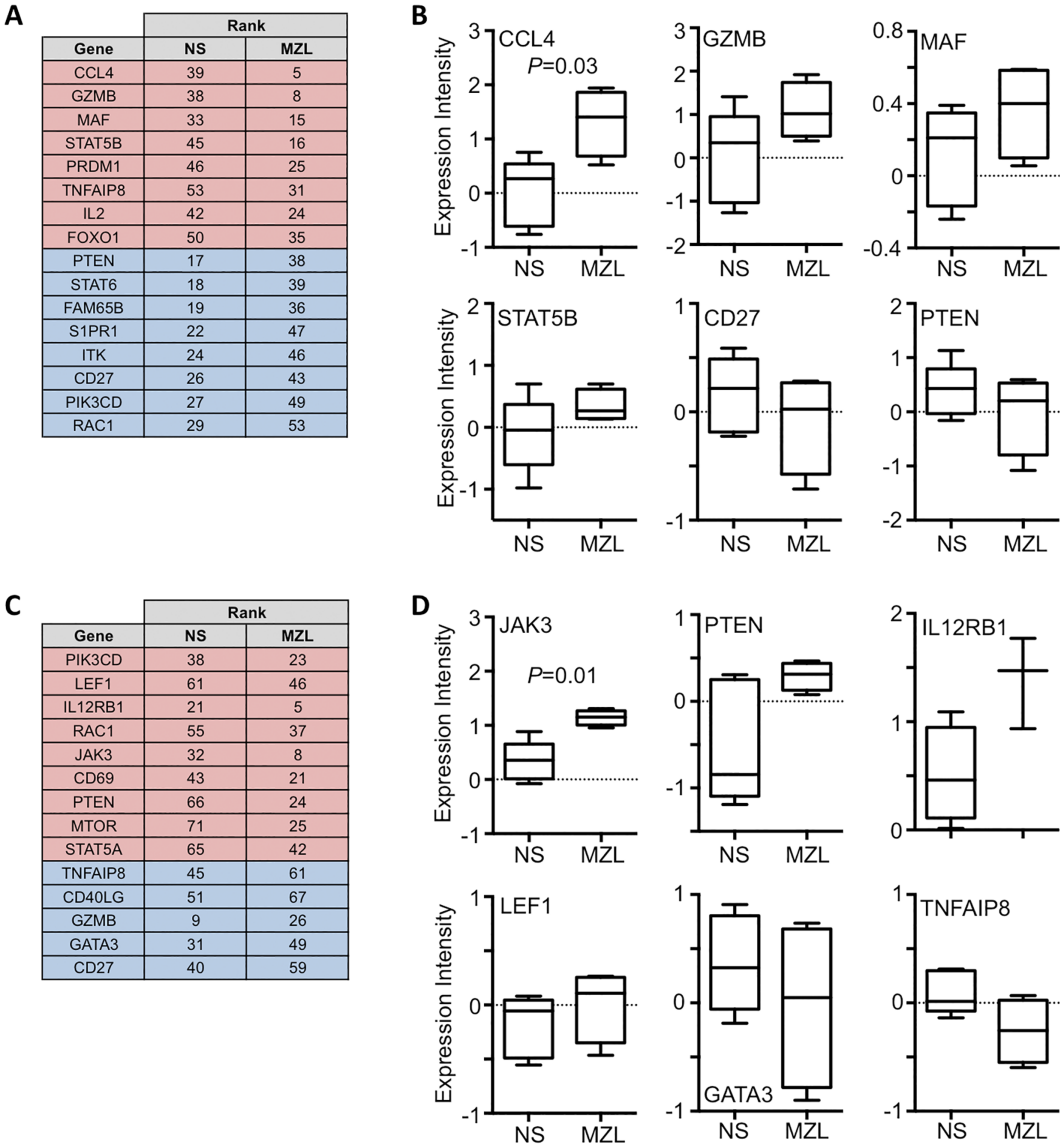
Gene expression changes between normal subjects and MZL patients. **(A)** Listing of genes showing the greatest relative change of expression level comparing cTfh PD1+ cells from normal subjects (NS) and patients with MZL. Genes showing an increase in rank in MZL i.e. increased expression are shaded red and those showing a decrease in rank are shaded blue. **(B)** Box and whisker plot (median, interquartile ranges and 10th and 90th percentile) showing expression levels of CCL4, which differed significantly between cTfh cells of normal subjects and MZL patients (Mann-Whitney U-test, *P* = 0.03). Examples of non-significant expression level changes are shown for GZMB, MAF, STAT5B, CD27 and PTEN. **(C)** Relative change in gene expression comparing cTfh1 PD1+ cells from normal subjects (NS) and patients with MZL. Genes showing an increase in rank in MZL i.e. increased expression are shaded red and those showing a decrease in rank are shaded blue. **(D)** Expression levels of JAK3 differed significantly (Mann-Whitney U-test, *P* = 0.01) between cTfh1 cells of normal subjects and MZL patients. Examples of non-significant expression level changes are shown for PTEN, IL12RB1, LEF1, GATA3 and TNFAIP8.

Overall cTfh PD1^+^ cell gene expression was similar in normal subjects and MZL patients (S2 Fig) but the chemokine, CCL4, was significantly (Mann-Whitney U-test, *P* = 0.03) elevated in MZL (Fig 6A). It is likely that the relatively few patients in our cohorts contributed to the lack of statistically significant differentially expressed genes. Therefore, we ordered genes with respect to their level of expression and compared ranks between normal subjects and MZL patients. Examples of individual genes whose expression is increased (GZMB, MAF and STAT5B) or decreased (CD27 and PTEN) in MZL patients is shown (Fig 6A). Within the cTfh1 PD-1^+^ subset, samples also clustered poorly (Fig 6B). JAK3 was significantly more highly expressed in MZL (*P* = 0.014) while other genes including IL12RB1, LEF1 and PTEN are increased and GATA3 and TNFAIP8 are decreased. The data suggests that circulating T-cell subsets from patients with lymphoma show patterns of gene expression that may allow distinction from the T-cells of normal subjects.

## Discussion

We report here the first analysis of cTfh in low-grade lymphomas focusing on MZL and FL. Our principal findings are 1) lymphoma patients demonstrate increased numbers of cTfh1 and reduced cTfh17 and 2) gene expression differences between normal subjects and patients are detectable in both bulk cTfh populations and cTfh1 cells.

We considered whether technical factors could be responsible for the results we observed. The proportion of cTfh (defined as CD4^+^CXCR5^+^) obtained in this work (median 29%) is higher than in the literature (e.g. median 11%[8] or 16% [30] or 14% [28]). This is most likely due to the improved properties of the fluorophores we employed (Table 2) as we used the same monoclonal antibody clone as many others to detect CXCR5[28,31,32]. Secondly, although there is little human data on the effects of aging on Tfh function[33] changes to cTfh fraction have been reported with age. However, the reported differences appear small and the literature is contradictory: although definitions of cTfh differ some authors show increased fraction of cTfh (but not of absolute number) with age[30] whereas others demonstrate reduced proportions [32]. For those normal subjects, who were >40 years old (3/12 (25%)) in our cohort, there was no significant difference in cTfh as a proportion of CD4^+^ T-cells as anticipated from the minor differences observed by others. Therefore, it is unlikely that the age distribution of normal subject and patient groups in our study influenced the significant differences that we report.

There are two lines of evidence that cTfh are derived from Tfh: firstly, patients with immunodeficiency syndromes caused by impaired Tfh formation, such as ICOS deficiency, show significantly reduced cTfh [34] and secondly, the frequency of activated (PD1^hi^) cTfh cells correlates with Tfh differentiation in an antigen-specific manner [18].

Recent work [35] investigated the functions of lymph node Tfh and cTfh cells in mice. While both subsets produced B-cell proliferation cTfh cells produced more growth factors and had a greater role in promoting germinal centers than lymph node Tfh. This raises the possibility that cTfh have specific functions. Functionally cTfh subsets are efficient (cTfh2 and cTfh17) or inefficient (cTfh1) helper cells[20,36]. There is now evidence of increased numbers of cTfh cells in several autoimmune conditions, most commonly cTfh1 cells are reduced while cTfh2 or cTfh17 or both cTfh2 and cTfh17 cells are increased as compared to normal subjects. However, it is recognised that higher PD1 expression associates with higher Tfh activity [18,20,28] and, both increased cTfh numbers and PD1 expression are associated with corresponding changes in disease activity [17,31,37,38]. Overall, therefore, efficient helper subsets often associate with overactive immunity and might also associate with high grade B-cell lymphoma[25,39]. In line with these findings we demonstrate increased proportion of activated cTfh subsets in lymphoma patients as compared to healthy subjects.

Surprisingly there is also an increased proportion of activated cTfh1 cells in patients. cTfh1 cells provide less efficient help to naive B-cells and it has been suggested that this might contribute to the poor efficacy of influenza vaccines [26,32], which are associated with a predominant cTfh1 response.

However, as yet there is no consensus on the function of cTfh cells and evidence is emerging that cTfh1 cells associate with the production of broad neutralising HIV-1 antibodies and can induce class switching to IgG3 (but not IgG1), to secrete cytokines that support B-cell proliferation and cause B-cell maturation[40]. A hypothesis for understanding cTfh1 function in low-grade B-cell lymphoma is that this subset might maintain or expand pre-existing B-cell responses but not prime new antigen specific B-cells[40] or naive B-cells[26].

It is relevant to speculate on the potential role of IFNγ, a Th1 cytokine, in low-grade B-cell lymphoma. IFNγ is present in the normal mouse germinal centre[41] where it is associated with class switching to IgG2a, and has also been detected in the FL microenvironment [42,43] possibly mostly in T-cells [44]. Th1 cells producing IFNγ have been shown to predominate in early extra-nodal marginal zone lymphoma[45]. Our data is compatible with this literature and suggests the idea that Tfh1 cells might be present in the microenvironment of low grade B-cell lymphomas and that *in vivo* IFNγ might be required for maintenance or proliferation of the lymphoma cells.

Further support for a role of IFNγ in lymphomagenesis comes from a study of Sjögren’s syndrome, an autoimmune condition associated with a high incidence of marginal zone lymphoma (probably >50-fold greater than for normal subjects). The prediction of in situ lymphoma development was associated with higher IFNγ expression [46]. B-cells are capable of responding to IFNγ through the IFNγ -R[47] and this mechanism has been shown to be important in driving B-cells in systemic lupus erythematosus (SLE), another autoimmune condition.

Are there alternative routes whereby IFNγ might exert its effects? B-cell activating factor (BAFF) is produced by activated monocytes, myeloid cells or T-cells and promotes the differentiation and proliferation of B-cells [48,49]. IFNγ production by T-cells is stimulated by BAFF[50] and it is, therefore, conceivable that IFNγ and BAFF act together to promote lymphoma proliferation. In support of this, one study showed that the majority (77/116, 78%) of B-cell lymphoproliferative disease expressed BAFF-R [51] while others [52] demonstrate that as well as BAFF-R B-cell lymphomas could also express the other BAFF receptors (BCMA and TACI)

Although effects of Tfh1 infiltration of lymphomas are difficult to predict they could be associated with elevated circulating levels of IFNγ, and this could be explored as a biomarker. There has been little work in this area but one prospective study showed that IFNγ levels (together with levels of IL-2 and ICAM) associate with the risk of developing lymphoma [53].

It has been suggested that cTfh subsets could serve as potential biomarkers for monitoring antibody responses in vaccinations and infections, and dysregulated antibody responses in autoimmune diseases[20]. We propose that determining cTfh subset proportions might be biomarkers of response to treatment in low-grade B-NHL. We sought gene expression changes that could potentially add to the usefulness of cTfh as biomarkers in low grade B-NHL. We demonstrate that there are significant differences in gene expression between normal subjects and lymphoma patients for both bulk cTfh and the cTfh1 subset. These genes include a chemokine (CCL4) and signaling molecule (JAK3). JAK3 enhances TBX21 binding to the *IFNG* promoter and is required for Th1 differentiation[54] and IFNγ production suggesting a molecular connection between our observations on cTfh1 numbers and gene expression profiling.

Our results suggest that abnormal interactions between lymphoma B-cells and T-cells in the TME of low-grade B-NHL might contribute to alterations in cTfh subset proportions and gene expression. These results have implications for understanding the biology of the TME. Data derived from cTfh subsets could potentially also be used to develop a clinically useful score to guide treatment decisions.

## Supporting information

S1 Fig Gene expression in tonsillar Tfh and non-Tfh cells. **(A)** Heat map showing gene expression levels (rows) in sorted Tfh cells (CD4^+^CXCR5^+^) (n = 2) and non-Tfh cells (CD4^+^CXCR5^-^) (n = 2). Arrow-heads indicate the position of genes whose fold-change in expression levels are indicated in (B). **(B)** Fold-change in expression levels of individual genes involved in Tfh function or differentiation in Tfh cells (red circles) and non-Tfh cells (blue circles). Hierarchical clustering was performed using Pearson correlation.
(TIF)

S2 Fig Comparison of gene expression between normal subjects and patients with MZL. Heat maps show gene expression levels (rows) from normal subjects (n = 5) and MZL patients (n = 4) in **(A)** cTfh PD1^+^ cells and **(B)** cTfh1 PD1^+^ cells. There are significant differences in gene expression between normal subjects and lymphoma patients for CCL4 and JAK3 as indicated by the arrow-heads. Hierarchical clustering was performed using Pearson correlation.
(TIF)

S1 Table Genes and oligonucleotide primer pairs employed in microfluidic RT-qPCR. (DOCX)
