# Supplementary figures and images for: Circulating Tfh1 (cTfh1) cell numbers and PD1 expression are elevated in low-grade B-cell non-Hodgkin’s lymphoma and cTfh gene expression is perturbed in marginal zone lymphoma
Source: PLoS One. 2018 Jan 2;13(1):e0190468. doi: 10.1371/journal.pone.0190468 (PMC5749831; doi:10.1371/journal.pone.0190468)

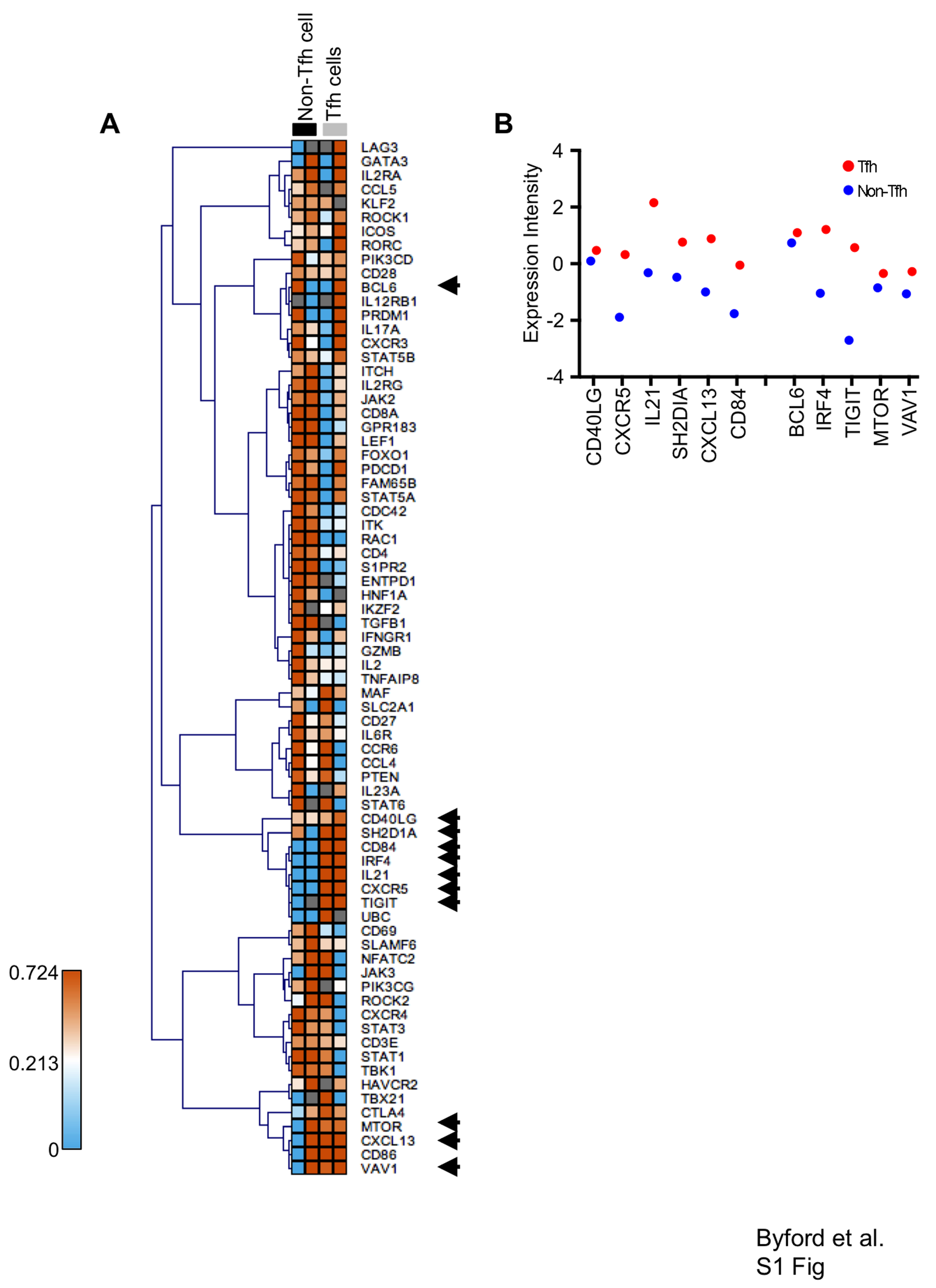

Supplement: S1 Fig — (A) Heat map showing gene expression levels (rows) in sorted Tfh cells (CD4+CXCR5+) (n = 2) and non-Tfh cells (CD4+CXCR5-) (n = 2). Arrow-heads indicate the position of genes whose fold-change in expression levels are indicated in (B). (B) Fold-change in expression levels of individual genes involved in Tfh function or differentiation in Tfh cells (red circles) and non-Tfh cells (blue circles). Hierarchical clustering was performed using Pearson correlation. (TIF) [file pone.0190468.s001.tif]

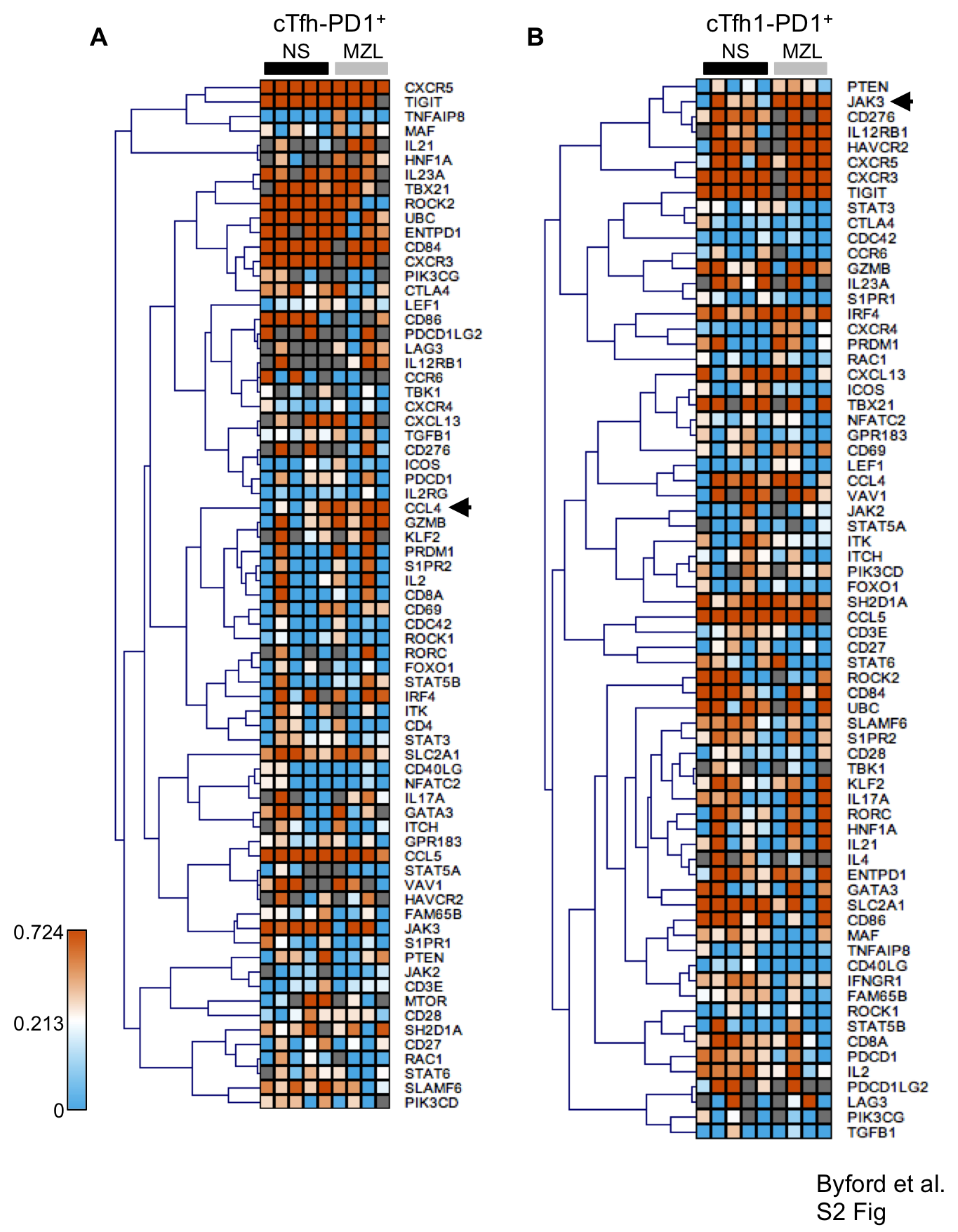

Supplement: S2 Fig — Heat maps show gene expression levels (rows) from normal subjects (n = 5) and MZL patients (n = 4) in (A) cTfh PD1+ cells and (B) cTfh1 PD1+ cells. There are significant differences in gene expression between normal subjects and lymphoma patients for CCL4 and JAK3 as indicated by the arrow-heads. Hierarchical clustering was performed using Pearson correlation. (TIF) [file pone.0190468.s002.tif]
