# Supplementary material for: Circulating Tfh1 (cTfh1) cell numbers and PD1 expression are elevated in low-grade B-cell non-Hodgkin’s lymphoma and cTfh gene expression is perturbed in marginal zone lymphoma
Source: PLoS One. 2018 Jan 2;13(1):e0190468. doi: 10.1371/journal.pone.0190468 (PMC5749831; doi:10.1371/journal.pone.0190468)
Supplement: S1 Table — (DOCX) [file pone.0190468.s003.docx]

**S1 Table**

**Genes and oligonucleotide primer pairs employed in microfluidic RT-qPCR.**

| **Gene symbol** | **Forward Primer** | **Reverse Primer** |
| --- | --- | --- |
| *ACTB* | CCAACCGCGAGAAGATGAC | TAGCACAGCCTGGATAGCAA |
| *ASCL2* | CTGGTGAACTTGGGCTTCCA | GAGCGCAGCGTCTCCA |
| *B2M* | TTAGCTGTGCTCGCGCTAC | CTCTGCTGGATGACGTGAGTAA |
| *BATF* | AGCAGTGACTCCAGCTTCA | CTCTTCTGGGCGGCAATAC |
| *BCL6* | GATGGAGCATGTTGTGGACAC | AGGAGGCTTGATGGCAGAAA |
| *CCL3* | GAGCAGCCAGTGCTCCAA | AGCTCCAGGTCGCTGACATA |
| *CCL4* | ACTATGAGACCAGCAGCCTCT | TCAGCACAGACTTGCTTGCT |
| *CCL5* | CCCTCGCTGTCATCCTCA | GGGCAATGTAGGCAAAGCA |
| *CCR6* | AGGCAGCGATGTCTGTGAA | AGCTCAAGCCCCAACATCA |
| *CD27* | CACTACTGGGCTCAGGGAAA | CCGGTATGCAAGGATCACAC |
| *CD274* | ACCAGCCGCGCTTCTGT | TCAGCAAATGCCAGTAGGTCATGAAT |
| *CD276* | GGGAGGGAGAAGGCTCCA | CTTCTTTGCTGTCAGAGTGTTTCA |
| *CD28* | GTGGAGTCCTGGCTTGCTATA | GAGCCTGCTCCTCTTACTCC |
| *CD3E* | GCAAACCAGAAGATGCGAAC | CATCACATCCATCTCCATGCA |
| *CD4* | AAAGTTGCATCAGGAAGTGAACC | CCCACACCTCACAGGTCAAA |
| *CD40LG* | GAGGCCAGCAGTAAAACAAC | AGTTGTTGCTCATGGTGTAGTA |
| *CD69* | TCACCCATGGAAGTGGTCAA | ACACACTTGTCAGACCCTGTA |
| *CD84* | TCCAAGGAAGAGCCAGTGAAC | GCTTGAAGTCCCAGGAGGTTTA |
| *CD86* | TCAGCTTGTCTGTTTCATTCCC | GCGTCTTGTCAGTTTCCAGAA |
| *CD8A* | CCATCATGTACTTCAGCCACTTCG | GCTGCGACGCGATGGT |
| *CDC42* | TCAGTGGTCTCTCCATCTTCA | TTGAGTCCCAACAAGCAAGAA |
| *CTLA4* | CATGGACACGGGACTCTACA | AATCTGGGTTCCGTTGCCTA |
| *CXCL13* | GGACCCTCAAGCTGAATGGATA | ACACTGGAACTGGTAGAGTTGAA |
| *CXCL9* | AGCCCTTCCTGCGAGAAAA | TCTGCTGAATCTGGGTTTAGACA |
| *CXCR3* | AACTGTGGCCGAGAAAGCA | TTGAGGCAGCAGTGCATGTA |
| *CXCR4* | CCCGACTTCATCTTTGCCAAC | ACACAACCACCCACAAGTCA |
| *CXCR5* | ATCTTCTTCCTCTGCTGGTCAC | GGTATTGTCCACGGCCTTCA |
| *ENTPD1* | CTGGGAGCACATCCATTTCA | GTCAGGTTCAGCATGTAGCC |
| *EOMES* | CTGTGGCAAAGCCGACAATA | CTCATCCAGTGGGAACCAGTA |
| *FAM65B* | TCAGCCTAAAAGGGTGGAAGAA | CGTCTGGTGAACCTCCAGATA |
| *FOXO1* | GGTGTCAGGCTGAGGGTTA | TTCTCTCAGTTCCTGCTGTCA |
| *FOXP3* | TGTGGGGTAGCCATGGAAA | GGGTCGCATGTTGTGGAA |
| *GAPDH* | GAACGGGAAGCTTGTCATCAA | ATCGCCCCACTTGATTTTGG |
| *GATA3* | CACGGTGCAGAGGTACCC | AGGGTAGGGATCCATGAAGCA |
| *GPR183* | ACAGAGACCCGAACGAGTCA | AGTTGCAGAGGGCGGAGTAA |
| *GZMB* | CTTCTCCAACGACATCATGCTAC | CTGGGCCTTGTTGCTAGGTA |
| *HAVCR2* | GGATCCAAATCCCAGGCATAA | CTTGGAAAGGCTGCAGTGAA |
| *HNF1A* | TGGTACGTCCGCAAGCA | ACCTGTGGGCTCTTCAATCA |
| *ICOS* | GCCAACTATTACTTCTGCAACC | GAACTTCAGCTGGCAACAAA |
| *IFNG* | ACTGCCAGGACCCATATGTAA | GTTCCATTATCCGCTACATCTGAA |
| *IFNGR1* | AAGCCAGGGTTGGACAAAA | GATATCCAGTTTAGGTGGTCCAA |
| *IKZF2* | AGGAAAGTCCAGGAGCTTCAA | AGAAGCTCCACACTGGTTACA |
| *IL10* | CCGTGGAGCAGGTGAAGAA | GTCAAACTCACTCATGGCTTTGTA |
| *IL12RB1* | GCCATATCCGGATGCAGAC | AGGAGCACTCGTAACGATCA |
| *IL17A* | ACTACAACCGATCCACCTCAC | ACTTTGCCTCCCAGATCACA |
| *IL2* | ACCCAGGGACTTAATCAGCAA | GCATATTCACACATGAATGTTGTTTCA |
| *IL21* | CTGAATTTCTGCCAGCTCCA | TTGTTTCCTGTATTTGCTGACTTTA |
| *IL21R* | TGCATCCTGGAAATGTGGAAC | CCTCGTCCTTCAGCTCTTCATA |
| *IL23A* | TCACAGAAGCTCTGCACAC | TCCACACTGGATATGGGGAA |
| *IL2RA* | TCCTGGGACAACCAATGTCA | GTCACTTGTTTCGTTGTGTTCC |
| *IL2RG* | GCCCAATGGGAATGAAGACA | TGGAAACACTGAGGGAGTCA |
| *IL4* | CAGCTGATCCGATTCCTGAAA | GTTGGCTTCCTTCACAGGAC |
| *IL6* | AGAGCTGTGCAGATGAGTACAA | GTTGGGTCAGGGGTGGTTA |
| *IL6R* | GTAGTGTCGGGAGCAAGTTCA | ATGTTGGCAGGCGGATCA |
| *IRF4* | CGGGCAAGCAGGACTACAA | TGTCGATGCCTTCTCGGAAC |
| *ITCH* | TGATGATGGCTCCAGATCCAA | ACCAGCTCCTGCATCTTCA |
| *ITK* | CAGCCGAGACAAAGCTGAA | TGCAGTCCTGGAATCCCTTA |
| *JAK2* | ACTTCTGCAGTACACATCTCA | CCAGATCCCTGTGGATATACC |
| *JAK3* | TCCTGTACGAGCTCTTCACCTA | CATCCCATCATCCGCAGGAA |
| *KLF2* | ATCCTGCCGTCCTTCTCCA | CCATGGACAGGATGAAGTCC |
| *LAG3* | TGGAGCCTTTGGCTTTCAC | GAGGGTGAATCCCTTGCTCTA |
| *LEF1* | AAGAAAGTGCAGCTATCAACCA | GCTGTCTTTCTTTCCGTGCTA |
| *MAF* | TCGACGACCGCTTCTCC | ATCACCTCCTCCTTGCTGAC |
| *MTOR* | CCAAACCCAGGTGTGATCAA | TCCTCATTTCCAGGCCACTA |
| *NFATC2* | TGGAAGCCACGGTGGATAA | TGTGCGGATATGCTTGTTCC |
| *PDCD1* | GCAGCCTGGTGCTGCTA | GTGCGCCTGGCTCCTA |
| *PDCD1LG2* | GGAATTGCAGCTTCACCAGATA | CACATTGCTGCCATGCTCTA |
| *PIK3CD* | GTGATTCAGCAGGGGAAGACTA | GGATGGTGTAGGCCCTTTCA |
| *PIK3CG* | TGGATCTATGCCTCCTGCCATA | TGTCGTGGCGTCTTTCACAA |
| *POU2AF1* | TCCGCCACTCATCACCAA | GGAGGTGGGTAGTGTGGAAA |
| *PRDM1* | CCTGGTACACACGGGAGAAAA | TTGAGATTGCTGGTGCTGCTA |
| *PTEN* | CCAGACATGACAGCCATCA | AGTCTTTCTGCAGGAAATCCC |
| *RAC1* | TCACCTATCCGCAGGGTCTA | GCCGAGCACTCCAGGTATTTTA |
| *ROCK1* | AGAAGCTGAACGAAGAGACA | TGCTTCACCTCCTCTTGTAAA |
| *ROCK2* | CCACTGAGAAAGTGAATCAACTCC | TTAACCGGGCTGCAGTATCA |
| *RORC* | CAAGACTCATCGCCAAAGCA | TTTCCACATGCTGGCTACAC |
| *S1PR1* | AAGCGAGCCGTACAGATCC | GAGAGGGCCTCGGAAACC |
| *S1PR2* | GACGCAGACGCCAAGG | GTGTTCCTGGACCTTGTTGG |
| *SH2D1A* | GAAGTCCTCAGCTAGAAGTACACA | GGGCTTTCAGGCAGACATCA |
| *SLAMF6* | CGGTTTCATCATACTGCTGTTAC | TGTGTTCGCTGAGTAGACAAA |
| *SLC2A1* | ATTGTGGGCATGTGCTTCC | AGAACCAGGAGCACAGTGAA |
| *STAT1* | ATGCTGGCACCAGAACGAA | GCTGGCACAATTGGGTTTCAA |
| *STAT3* | GGAAATAATGGTGAAGGTGCTGAAC | CCGAGGTCAACTCCATGTCAAA |
| *STAT4* | CAGTGCTGGAGGTAAAGGAA | AGAGGCAGATCTGTGTTTCAA |
| *STAT5A* | CCCAGGCTCCCTATAACATGTA | ATGGTCTCATCCAGGTCGAA |
| *STAT5B* | AACAGAGGTTGGTCCGAGAA | GTTTCTGGGACATGGCATCA |
| *STAT6* | TGTCCTGGTCGCAGTTCA | TCCAGGACACCATCAAACCA |
| *SYK* | AGCACTGCGTGCTGATGAA | TGATGCATTCCGGAGCGTAC |
| *TBK1* | AGATGGAAGGGGTGGTTAAAGAA | AGGCCACCATCCATGGTTAA |
| *TBX21* | GGGCGTCCAACAATGTGAC | CCGTCGTTCACCTCAACGATA |
| *TGFB1* | CGTCTGCTGAGGCTCAAGTTA | TCGCCAGGAATTGTTGCTGTA |
| *TIGIT* | GTGGTGGTCGCGTTGACTA | TCCTGTCCAGCTGATTTTCTCC |
| *TNFAIP8* | ACCAGGGAGTACACCCAAAA | AGAATGGCCAGCTTGATGAC |
| *TNFRSF18* | GCTGCTGCCGCGATTA | GAATTCAGGCTGGACACACA |
| *UBC* | TCGGCCTTAGAACCCCAGTA | GAAAACCAGTGCCCTAGAGTCA |
| *VAV1* | ATCAACCTGCGTGAGGTCAA | CAGGTGGACAGGAAGGTTCTAA |
